# Alzheimer's Disease and Hippocampal Adult Neurogenesis; Exploring Shared Mechanisms

**DOI:** 10.3389/fnins.2016.00178

**Published:** 2016-05-03

**Authors:** Carolyn Hollands, Nancy Bartolotti, Orly Lazarov

**Affiliations:** Department of Anatomy and Cell Biology, College of Medicine, The University of Illinois at ChicagoChicago, IL, USA

**Keywords:** hippocampus, neurogenesis, Alzheimer's disease, cognition, learning and memory

## Abstract

New neurons incorporate into the granular cell layer of the dentate gyrus throughout life. Neurogenesis is modulated by behavior and plays a major role in hippocampal plasticity. Along with older mature neurons, new neurons structure the dentate gyrus, and determine its function. Recent data suggest that the level of hippocampal neurogenesis is substantial in the human brain, suggesting that neurogenesis may have important implications for human cognition. In support of that, impaired neurogenesis compromises hippocampal function and plays a role in cognitive deficits in Alzheimer's disease mouse models. We review current work suggesting that neuronal differentiation is defective in Alzheimer's disease, leading to dysfunction of the dentate gyrus. Additionally, alterations in critical signals regulating neurogenesis, such as presenilin-1, Notch 1, soluble amyloid precursor protein, CREB, and β-catenin underlie dysfunctional neurogenesis in Alzheimer's disease. Lastly, we discuss the detectability of neurogenesis in the live mouse and human brain, as well as the therapeutic implications of enhancing neurogenesis for the treatment of cognitive deficits and Alzheimer's disease.

## Introduction

In early development neurons are rapidly produced to form the intricate complexity of the brain and peripheral nervous system. Postnatally, the role of neurogenesis is shifted from brain development into brain plasticity. From then on, neurogenesis takes place only in specific niches in the adult brain, in the subgranular zone (SGZ) of the dentate gyrus (DG) of the hippocampus and the subventricular zone (Kempermann et al., 2015). Recent evidence suggests substantial levels of hippocampal neurogenesis in the human brain, estimating about 700 new neurons a day in the DG (Spalding et al., 2013). Humans replace ~35% of the DG, while rodents are estimated to replace only 10% (Ninkovic and Gotz, 2007; Imayoshi et al., 2008). Recent information also suggests that in humans, the striatum may be a source of adult neurogenesis as well (Ernst et al., 2014). The existence of adult neurogenesis in the human brain supports the notion that neurogenesis has important functional significance and implications for cognitive disorders and their therapy (Eriksson et al., 1998; Ninkovic and Gotz, 2007; Imayoshi et al., 2008; Lazarov and Marr, 2013; Spalding et al., 2013).

**Figure 1:**
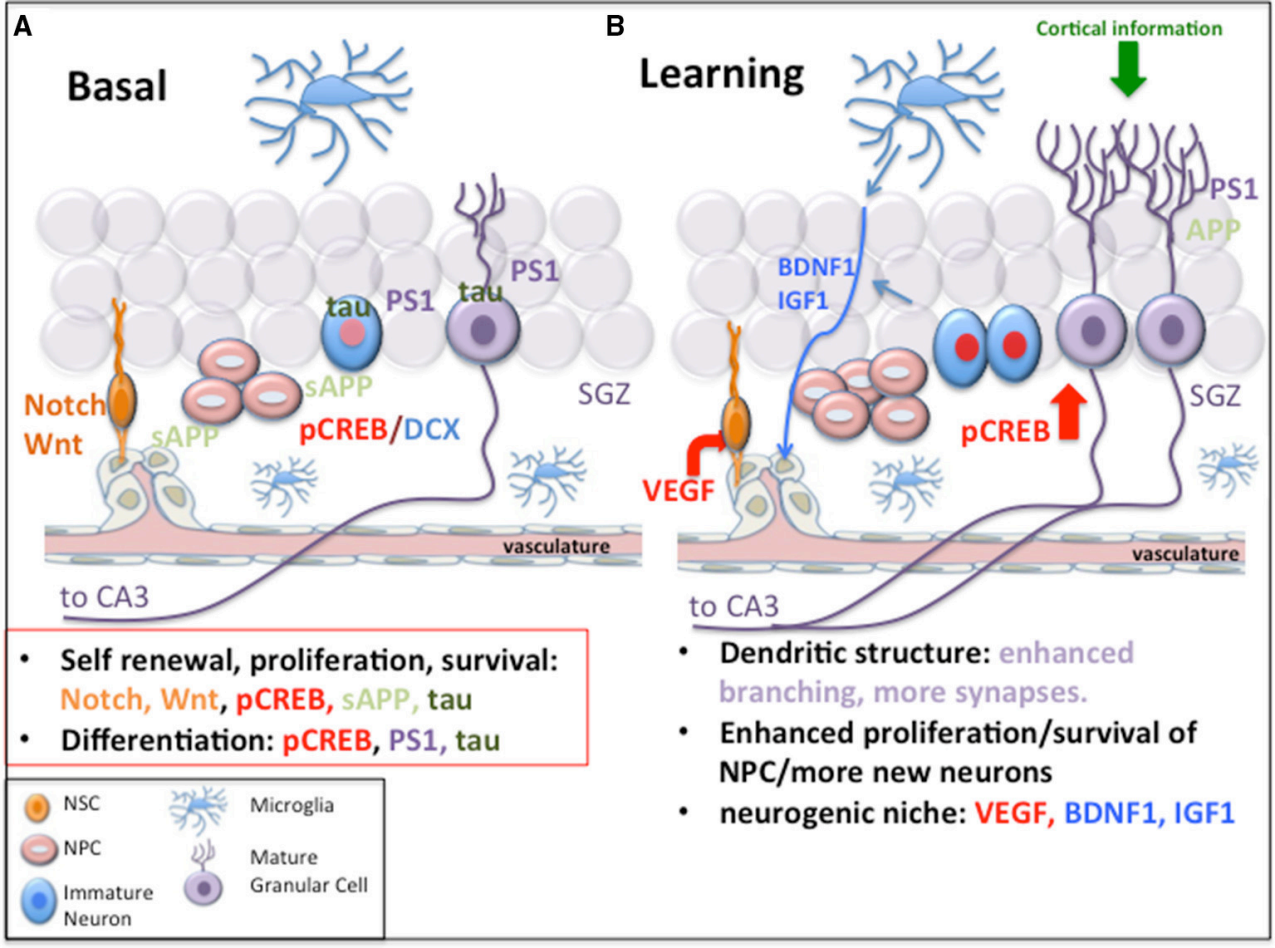
**Common mechanisms of neurogenesis and Alzheimer's disease and the implications for learning**. **(A)** Signals that play a role in neurogenesis, such as Notch-1, Wnt/β-catenin, CREB, sAPP, tau, and presenilin-1 are implicated in Alzheimer's disease. **(B)** Following learning, changes in the neurogenic niche include alterations in Notch and sAPP, increased expression of neurotrophins such as VEGF, BDNF, and IGF which enhance angiogenesis and provide support for the neurogenic niche and lead to increased neurogenesis. Upregulation of CREB signaling by neural progenitor cells and neurons may promote survival and maturation of NPCs. Increased dendritic branching of mature neurons and synaptic plasticity may be mediated by presenilin-1 and APP. The factors mediating these processes are dysfunctional or compromised in Alzheimer's disease, suggesting that defective neurogenesis may affect hippocampal function in Alzheimer's disease.

The circuitry of the DG, of which new neurons are part, promotes several important functions, namely, pattern separation, conjunctive encoding of multiple sensory output to the dorsal CA3, facilitation of encoding of spatial information based on its output to the dorsal CA3, and encoding of time in new memories (for review, Lazarov and Hollands, 2016). In support of the role of hippocampal neurogenesis in plasticity, learning and memory, increasing evidence suggests that cognitive deficits, difficulty learning new information and memory loss, as occurs in Alzheimer's disease (AD), may be, at least in part, due to impairments in adult neurogenesis (Demars et al., 2010, 2013; Lazarov and Marr, 2010; Lazarov et al., 2010). Some of the foundation for the association between impairments in adult hippocampal neurogenesis and cognitive deficits leading to AD might be due to the fact that several key signals implicated in AD play a role in regulation of hippocampal neurogenesis (Figure 1).

## Neurogenesis in aging, disease state, and cognitive dysfunction

In the rodent brain, neurogenesis is dramatically decreased during adulthood and further declines during aging (Demars et al., 2013). Recent evidence suggests that in wild type mice reduced proliferation of neural progenitor cells (NPCs) might be one of the processes underlying this phenomenon (Demars et al., 2013). However, other mechanisms, such as altered signaling, increased quiescence of neural stem cells (NSCs) and differentiation toward non-neuronal subtypes have been proposed [see for example Hattiangady and Shetty, 2008]. In humans, the dynamics are less clear. A recent study suggests that there is a moderate decline in neurogenesis with aging (Spalding et al., 2013). However, as of yet, it is unclear how this decline impacts cognitive function in humans or whether similar memory paradigms are regulated by adult neurogenesis as they are in rodents. Observations in humans using high resolution fMRI (Brickman et al., 2014) and cognitive studies (Toner et al., 2009; Stark et al., 2010; Yassa et al., 2011; Brickman et al., 2012) suggest that age-related memory loss begins in the DG. These changes are believed to stem from a decline in the support of the neurogenic niche as well as intrinsic characteristics of NSC (for review Silva-Vargas et al., 2013). Many processes decline in the aging brain along with a decrease in adult neurogenesis. For example, in both rodents and humans the density of synaptic contacts onto granular cells in the DG decreases with age (Flood et al., 1996; Geinisman et al., 2001, 2004). It will be important to determine whether age-dependent decline in neurogenesis compromises the function of the DG and induces susceptibility to memory impairments.

Deficits in adult neurogenesis with age may compromise the structure and function of the entorhinal-hippocampal circuit. This area is particularly vulnerable and heavily affected in AD, the most common form of dementia. AD is characterized by progressive memory loss and cognitive dysfunction (Baulac et al., 2003). Rare, Familail AD (FAD) is caused by mutations in the *amyloid precursor protein* (*APP*) and *presenilin 1* and *2* (*PS1,2*) (Selkoe and Wolfe, 2007). However, the majority of AD cases are sporadic and aging is the greatest risk factor for AD. Research done in mouse models of FAD suggests that declining neurogenesis is an early stage event that can be observed as early as 2–3 months of age (Rodriguez et al., 2008; Demars et al., 2010; Hamilton et al., 2010)(for review Lazarov and Marr, 2010, 2013). Nevertheless, it is important to note that some FAD mouse lines, mostly lines that overexpress APP, exhibit enhanced, rather than reduced, neurogenesis (Jin et al., 2004; Chuang, 2010). As discussed below, this might be attributed to the overexpression of soluble APP (sAPP), a proliferation factor of NPCs (Demars et al., 2011, 2013; Lazarov and Demars, 2012). The manifestations of neurogenic impairments in FAD mice are diverse. They include defective maturation/reduced rate of survival of new neurons in the granular cell layer (GCL), compromised dendritic tree branching (Sun et al., 2009; Bonds et al., 2015), imbalance of GABAergic and glutamatergic input onto new granular neurons (Sun et al., 2009), expression of the less potent proliferation factor sAPPβ at the expense of sAPPα in the neurogenic niche (Demars et al., 2011, 2013) and loss of γ-secretase function in NPCs and new neurons (Gadadhar et al., 2011; Bonds et al., 2015).

Below, we highlight several key signaling factors that are implicated in AD and were recently described to regulate neurogenesis. These factors play a role in aging-dependent behavior, circadian rhythm, inflammation, oxidative stress, neurotrophic signaling, hormonal signaling, neurotransmission, vascular signaling, and others. Thus, the multi- factorial effect on neurogenesis exposes the complex relationship between neurogenesis and the progression of AD pathology (for review Lazarov and Marr, 2010, 2013; Lazarov et al., 2010).

## Alterations in molecular signals during aging and cognitive dysfunction accompanying neurodegenerative disease

*Presenillin-1* (PS1) is the catalytic core of γ-secretase, an aspartyl protease, which cleaves numerous substrates, including APP and Notch (De Strooper et al., 1998, 1999). Mutations in PS1 cause FAD, presumably due to loss of γ-secretase function (Xia et al., 2015). A recent paper suggests that PS1 undergoes a conformational change during aging and sporadic AD, and this change may have downstream effects on the processing of its substrates APP and Notch (Wahlster et al., 2013). PS1 regulates NPC differentiation in the adult brain (Gadadhar et al., 2011) via β-catenin, Notch1 and CREB (Bonds et al., 2015). Down regulation of PS1 in hippocampal NPCs compromises the maturation of new neurons, manifested by deficits in their dendritic tree branching, leading to learning and memory deficits (Bonds et al., 2015), suggesting that PS1-induced dysfunction of neurogenesis can impair cognitive function in AD. Transgenic expression of FAD-linked mutant variants of PS1 also impairs neurogenesis and the neurogenic response to experience in an enriched environment (EE) (Wang et al., 2004; Wen et al., 2004; Chevallier et al., 2005; Choi et al., 2008).

*Amyloid precursor protein (APP)*- APP is a substrate of γ-secretase. Misregulated cleavage of APP in the amyloidogenic pathway is implicated in FAD. While the physiological role of APP is yet to be fully understood, numerous studies suggest a role in synaptic plasticity and neurogenesis (Lazarov and Demars, 2012). The soluble form of APP (sAPPα) regulates NPC proliferation and survival (Demars et al., 2011, 2013). In fact, neurogenesis can be upregulated in the aging mouse brain following injection of sAPPα into the SVZ (Demars et al., 2013). While APP is extensively researched in regards to AD, the regulation of APP with aging is less well studied. However, there is some evidence that APP processing may be altered during aging, perhaps through dysregulation of the circadian system (Dobrowolska et al., 2014). In FAD, there is upregulation of the less potent sAPPβ counterpart at the expense of sAPPα, which may compromise proliferation of NPCs (Demars et al., 2011, 2013). Interestingly, sAPPα plays an important role in migration of NPC during brain development (Young-Pearse et al., 2007, 2008). Other metabolites of APP, such as AICD and Aβ have been suggested to regulate neurogenesis (for review see Lazarov et al., 2010), but more studies are warranted in order to establish their role.

*Tau*- is a neuronal microtubule-associated protein, the hyperphosphorylation and aggregation of which plays a key role in AD pathology. Significantly, adult born neurons transiently express the tau-3R isoform during development, overlapping with DCX and NeuN co-expression in the DG (Bullmann et al., 2007; Llorens-Martin et al., 2012). Tau phosphorylation in the DG is also temporally and spatially linked to DCX and neuroD expression with activated GSK-β believed to be the main tau kinase in newborn neurons (Fuster-Matanzo et al., 2009; Hong et al., 2010). The genomic based hTau mouse model exhibited reduction in adult neurogenesis, as a result of decreased proliferation, as early as 2 months of age before the appearance of significant tau pathology (Komuro et al., 2015), which may suggest that either impaired hippocampal neurogenesis is an early hallmark of tau pathology in AD or that there is an association between tau pathology and defective neurogenesis in AD. For a comprehensive review about tau and adult neurogenesis see (Fuster-Matanzo et al., 2012).

*Notch 1*- is a critical neurogenic signal and a substrate of γ-secretase. The intracellular domain cleavage product, NICD, translocates to the nucleus and drives transcription of factors important for maintaining the NSC pool such as *Hes* and *ErbB2* (for review Pierfelice et al., 2011). Notch signaling occurs when the Notch receptor is activated by one of its ligands in the Jagged or Delta-like family of proteins (for review Kopan and Ilagan, 2009). Following physical activity, NPC proliferation is increased in a Notch-dependent manner in the SGZ of the DG, even in aged mice (Lugert et al., 2010). In contrast, Notch signaling is decreased with age, including in the hippocampus (Lugert et al., 2010; Tseng et al., 2014). Down regulation of PS1 in hippocampal NPC results in reduced levels of NICD (Bonds et al., 2015). In mature neurons Notch levels are low, and its function is not fully elucidated (for review see Marathe and Alberi, 2015; Marathe et al., 2015).

*Wnt/*β*-catenin*- are critical signaling factors in the regulation of hippocampal neurogenesis (Chenn and Walsh, 2003; Sato et al., 2004; Lie et al., 2005; Shimizu et al., 2008). Wnt3 is expressed in the SGZ of the DG, and overexpression of Wnt3 is sufficient to increase neurogenesis (Lie et al., 2005). Wnts are produced by astrocytes in the adult hippocampal niche and support the proliferation and differentiation of neuronally-restricted NPCs (Lie et al., 2005). Wnts regulate NSC self-renewal by inactivating Glycogen synthase kinase 3 (GSK3) and stabilizing β-catenin (Shimizu et al., 2008). Further, β-catenin promotes NPC proliferation through the activation of LEF/TCF transcription factors (Shimizu et al., 2008). Interestingly, nuclear accumulated β-catenin also induces anti-neurogenic hes1 gene expression through the enhancement of Notch1- and RBP-J-mediated transcription. β-catenin can associate with the NICD, and it is present in a nuclear protein-DNA complex containing the hes1 gene promoter. The β-catenin–NICD complex is efficiently formed when transcriptional coactivators p300 and P/CAF are present. Also, significantly, following its cleavage, the PS1CTF/NTF forms a complex with GSK3 and β-catenin (Tesco et al., 1998; Tesco and Tanzi, 2000). PS1 has been implicated as a negative regulator of the Wnt/β-catenin signaling pathway (Xia et al., 2001). Wnt-independent interaction of β-catenin and PS1 has also been described (Kang et al., 2002). Downregulation of PS1 in adult NPCs compromises the phosphorylation of β-catenin, which may affect β-catenin translocation to the nucleus, leading to alterations in the normal development of NPC (Bonds et al., 2015).

*CREB*- Cyclic-AMP Response Element Binding protein (CREB) is a critical signaling factor for adult brain plasticity and learning (for review Kandel, 2012). Activation of CREB by phosphorylation on Ser133 (pCREB) is observed in the hippocampus and cortical areas following learning and memory tasks (for review Mayr and Montminy, 2001). Importantly, NPCs, neuroblasts and immature neurons constitutively express pCREB, suggesting that pCREB is a critical component of neurogenesis. Indeed, CREB plays a role in neuronal maturation and survival in hippocampal neurogenesis (for review Ge et al., 2006; Jagasia et al., 2009; Herold et al., 2011; Merz et al., 2011). In rodents, CREB signaling components in the hippocampus decrease with age (Chung et al., 2002; Kudo et al., 2005; Porte et al., 2008). However, these observations were made primarily in mature neurons. Thus, the impact of aging on NPC-specific CREB signaling remains unclear. Also unclear is how aging causes a decrease in CREB signaling, although hypotheses suggest that this could occur either by aging-dependent increased levels of reactive oxygen species, or via decreased NMDA receptor and BDNF expression, which are both important for CREB activation (Chung et al., 2002; Kudo et al., 2005; Porte et al., 2008; Ozgen et al., 2010). Interestingly, exposure to young blood increased CREB activation and neurogenesis in the aged hippocampus, suggesting that systemic factors that are altered with aging may play an important role in CREB signaling and neurogenesis in the brain (Villeda et al., 2011; Villeda and Wyss-Coray, 2013). Impaired CREB signaling in AD has been the subject of much study. CREB signaling is dysregulated in both human AD and in mouse models of FAD (Vitolo et al., 2002; Ma et al., 2007; Caccamo et al., 2010; Bartolotti et al., 2015). In addition, down regulation of PS1 expression in NPCs compromises pCREB expression, leading to defective maturation of new neurons and induction of cognitive deficits (Bonds et al., 2015). While the role of CREB signaling in memory via mature neurons is well documented, the contribution of CREB signaling in NPCs to memory is not fully elucidated, and separating out the contribution of CREB to learning and memory via mature neurons or via NPC function is technically challenging and remains to be investigated (for review see Scott Bitner, 2012; Ortega-Martinez, 2015). Likewise, most of the work on CREB signaling in AD has focused on the transient activation in mature neurons during the formation of long-term memories, and so the contribution of CREB signaling in NPC in the context of AD also remains an open question.

## Neurogenesis as a biomarker of cognitive function and as a therapeutic approach

While it is clear that hippocampal neurogenesis takes place in the human brain and that the number of new neurons generated is significant (Spalding et al., 2013), information concerning the fate of neurogenesis in aging and cognitively impaired individuals is scarce. Current techniques allow the examination of neurogenesis postmortem. However, because of the dynamic modulation neurogenesis can undergo following numerous stimuli, such as progressive pathology, the development of methodologies for the detection of neurogenesis in live individuals will be crucial. Up to the present time, tools for the detection of neurogenesis in live humans have been limited. The level of ^14^C in genomic DNA has been used for the estimation of date of birth of hippocampal neurons and their quantification in postmortem tissue (Spalding et al., 2013). A previous study suggests that adult neurogenesis can be specifically detected by proton nuclear magnetic resonance spectroscopy (^1^H-MRS, Manganas et al., 2007). However, this method was challenged by Loewenbruck et al. (2011), thus, more studies are warranted for the determination of the specificity, sensitivity and feasibility of ^1^H-MRS for the detection and quantification of neurogenesis.

**Figure 2:**
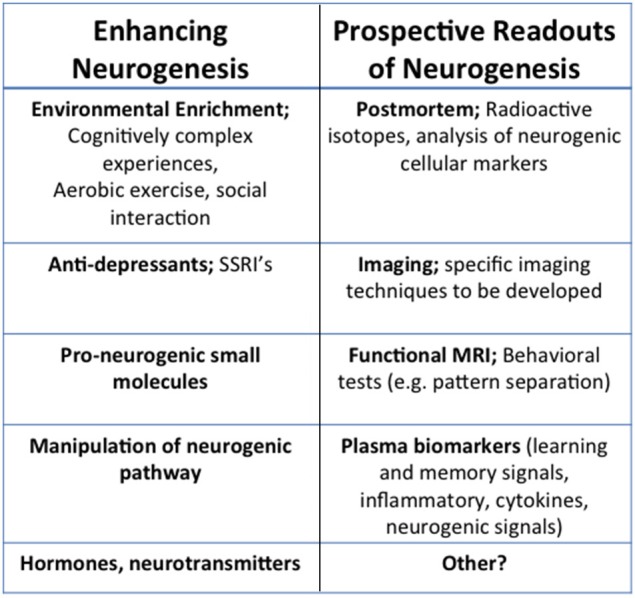
**Therapeutic and translational potential of neurogenesis**. Examples of current and prospective methods for the modulation and detection of neurogenesis. Means of enhancing neurogenesis include noninvasive, environmental modulations like cognitively complex activities and exercise, as well as molecular interventions like anti-depressants, pro-neurogenic small molecules, hormones or neurotransmitters, or other manipulations of the neurogenic pathways. While readouts of human neurogenesis are typically done in postmortem tissue using radioactive isotopes or analysis of neurogeneic cell markers, imaging techniques such as fMRI, or blood biomarkers will offer non-invasive avenues to determine neurogenesis during life.

The association between decline in neurogenesis and cognitive deterioration during aging, coupled with disruption in neurogenesis and cognitive dysfunction in FAD mouse models suggests that enhancing neurogenesis may be a feasible therapeutic approach (Figure 2). Successful attempts to enhance neurogenesis in rodents have been described. For example Sahay et al. used genetic manipulation of neurogenic pathways, excising the pro-apoptotic gene *Bax*, to enhance survival of nestin expressing cells (Sahay et al., 2011). They observed enhanced performance in the DG-dependent pattern separation task, where animals must distinguish between two similar contexts. Wang et al. also enhanced cell survival, neuronal differentiation, and dendritic complexity in neurogenic regions through activation of ERK5 map kinase (Wang et al., 2014). Following this manipulation, animals had increased performance in spatial learning and memory in the Morris Water Maze (MWM) task. In MWM and the novel object recognition task they also probed long-term memory and saw improvements as well, suggesting that adult neurogenesis may be a key therapeutic target.

Given the evidence from genetic manipulation of neurogenesis in rodents, it is important to consider how neurogenesis could be modulated in humans. One approach is the modulation of lifestyle factors, termed environmental enrichment (EE). Evidence from rodents suggests that EE and running are effective ways to enhance hippocampal plasticity and neurogenesis in particular (Kempermann et al., 1997; van Praag et al., 1999a,b). These behavioral interventions have been found to enhance neurogenesis and ameliorate pathology in AD mouse models (Lazarov et al., 2005; Lazarov and Larson, 2007; Hu et al., 2010, 2013). Significantly, studies have shown that exercise can improve cognitive performance in the elderly (Ahlskog et al., 2011). Brief increases in physical activity (6–12 months) upregulates hippocampal volume and improves both episodic and spatial memory (Klusmann et al., 2010; Erickson et al., 2011; Ruscheweyh et al., 2011). In rodents it has also been shown that EE can increase many of the molecular factors involved in neurogenesis, such as pCREB expression and CRE- gene transcription in the hippocampus of wild-type mice (Hu et al., 2013; Bartolotti et al., 2015). While this observation was not specific to new neurons, it raises the possibility that enhanced CREB signaling may be one mechanism by which EE may increase the survival of new neurons. Nevertheless, EE and running do not target neurogenesis specifically, but have numerous effects on the hippocampus. Several studies describe the manipulation of neurogenesis using small molecules (Longo et al., 2006; Schneider et al., 2008; McNeish et al., 2010; Pieper et al., 2010; Lange et al., 2011; MacMillan et al., 2011; Neely et al., 2012; Petrik et al., 2012; Shi et al., 2013) or pharmacological agents, such as SSRI's or modulators of neurogenic pathways [For example, see Warner-Schmidt and Duman, 2007]. Some of these have been shown to enhance neurogenesis and reverse memory deficits. However, to this point the use of these compounds in AD mouse models has not been explored. In future experiments it will be important to consider the mechanism by which these molecules modulate adult neurogenesis in light of the signaling cascades we have described here. Particularly considering how these cascades are altered in aging and AD, both in rodent models and in humans.

## Author contributions

This manuscript is based on data produced by Dr. CH and Mrs. NB. Dr. CH and Ms. NB and Prof. OL wrote this mini-review.

### Conflict of interest statement

The authors declare that the research was conducted in the absence of any commercial or financial relationships that could be construed as a potential conflict of interest.

## References

[B1] Ahlskog J. E. Geda Y. E. Graff-Radford N. R. Petersen R. C. (2011). Physical exercise as a preventive or disease-modifying treatment of dementia and brain aging. Mayo Clin. Proc. 86, 876–884. 10.4065/mcp.2011.0252 21878600 PMC3258000

[B2] Bartolotti N. Segura L. Lazarov O. (2015). Diminished CRE-induced plasticity is linked to memory deficits in familial Alzheimer's Disease mice. J. Alzheimers Dis. 50, 477–489. 10.3233/JAD-150650 26682682 PMC4927858

[B3] Baulac S. LaVoie M. J. Kimberly W. T. Strahle J. Wolfe M. S. Selkoe D. J.. (2003). Functional gamma-secretase complex assembly in Golgi/trans-Golgi network: interactions among presenilin, nicastrin, Aph1, Pen-2, and gamma-secretase substrates. Neurobiol. Dis. 14, 194–204. 10.1016/S0969-9961(03)00123-2 14572442

[B4] Bonds J. A. Kuttner-Hirshler Y. Bartolotti N. Tobin M. K. Pizzi M. Marr R.. (2015). Presenilin-1 dependent neurogenesis regulates hippocampal learning and memory. PLoS ONE 10:e0131266. 10.1371/journal.pone.0131266 26098332 PMC4476567

[B5] Brickman A. M. Khan U. A. Provenzano F. A. Yeung L. K. Suzuki W. Schroeter H.. (2014). Enhancing dentate gyrus function with dietary flavanols improves cognition in older adults. Nat. Neurosci. 17, 1798–1803. 10.1038/nn.3850 25344629 PMC4940121

[B6] Brickman A. M. Meier I. B. Korgaonkar M. S. Provenzano F. A. Grieve S. M. Siedlecki K. L.. (2012). Testing the white matter retrogenesis hypothesis of cognitive aging. Neurobiol. Aging 33, 1699–1715. 10.1016/j.neurobiolaging.2011.06.001 21783280 PMC3222729

[B7] Bullmann T. de Silva R. Holzer M. Mori H. Arendt T. (2007). Expression of embryonic tau protein isoforms persist during adult neurogenesis in the hippocampus. Hippocampus 17, 98–102. 10.1002/hipo.20255 17183532

[B8] Caccamo A. Maldonado M. A. Bokov A. F. Majumder S. Oddo S. (2010). CBP gene transfer increases BDNF levels and ameliorates learning and memory deficits in a mouse model of Alzheimer's disease. Proc. Natl. Acad. Sci. U.S.A. 107, 22687–22692. 10.1073/pnas.1012851108 21149712 PMC3012497

[B9] Chenn A. Walsh C. A. (2003). Increased neuronal production, enlarged forebrains and cytoarchitectural distortions in beta-catenin overexpressing transgenic mice. Cereb. Cortex 13, 599–606. 10.1093/cercor/13.6.599 12764034

[B10] Chevallier N. L. Soriano S. Kang D. E. Masliah E. Hu G. Koo E. H. (2005). Perturbed neurogenesis in the adult hippocampus associated with presenilin-1 A246E mutation. Am. J. Pathol. 167, 151–159. 10.1016/S0002-9440(10)62962-8 15972961 PMC1603433

[B11] Choi S. H. Veeraraghavalu K. Lazarov O. Marler S. Ransohoff R. M. Ramirez J. M.. (2008). Non-cell-autonomous effects of presenilin 1 variants on enrichment-mediated hippocampal progenitor cell proliferation and differentiation. Neuron 59, 568–580. 10.1016/j.neuron.2008.07.033 18760694 PMC3489017

[B12] Chuang T. T. (2010). Neurogenesis in mouse models of Alzheimer's disease. Biochim. Biophys. Acta 1802, 872–880. 10.1016/j.bbadis.2009.12.008 20056145

[B13] Chung Y. H. Kim E. J. Shin C. M. Joo K. M. Kim M. J. Woo H. W.. (2002). Age-related changes in CREB binding protein immunoreactivity in the cerebral cortex and hippocampus of rats. Brain Res. 956, 312–318. 10.1016/S0006-8993(02)03562-X 12445700

[B14] Demars M. Hu Y. S. Gadadhar A. Lazarov O. (2010). Impaired neurogenesis is an early event in the etiology of familial Alzheimer's disease in transgenic mice. J. Neurosci. Res. 88, 2103–2117. 10.1002/jnr.22387 20209626 PMC3696038

[B15] Demars M. P. Bartholomew A. Strakova Z. Lazarov O. (2011). Soluble amyloid precursor protein: a novel proliferation factor of adult progenitor cells of ectodermal and mesodermal origin. Stem Cell Res. Ther. 2, 36. 10.1186/scrt77 21878106 PMC3219067

[B16] Demars M. P. Hollands C. Zhao K. D. Lazarov O. (2013). Soluble amyloid precursor protein-alpha rescues age-linked decline in neural progenitor cell proliferation. Neurobiol. Aging. 34, 2431–2440. 10.1016/j.neurobiolaging.2013.04.016 23683827 PMC3706568

[B17] De Strooper B. Annaert W. Cupers P. Saftig P. Craessaerts K. Mumm J. S.. (1999). A presenilin-1-dependent gamma-secretase-like protease mediates release of Notch intracellular domain. Nature 398, 518–522. 10.1038/19083 10206645

[B18] De Strooper B. Saftig P. Craessaerts K. Vanderstichele H. Guhde G. Annaert W.. (1998). Deficiency of presenilin-1 inhibits the normal cleavage of amyloid precursor protein. Nature 391, 387–390. 10.1038/34910 9450754

[B19] Dobrowolska J. A. Michener M. S. Wu G. Patterson B. W. Chott R. Ovod V.. (2014). CNS amyloid-beta, soluble APP-alpha and -beta kinetics during BACE inhibition. J. Neurosci. 34, 8336–8346. 10.1523/JNEUROSCI.0540-14.2014 24920637 PMC4051982

[B20] Erickson K. I. Voss M. W. Prakash R. S. Basak C. Szabo A. Chaddock L.. (2011). Exercise training increases size of hippocampus and improves memory. Proc. Natl. Acad. Sci. U.S.A. 108, 3017–3022. 10.1073/pnas.1015950108 21282661 PMC3041121

[B21] Eriksson P. S. Perfilieva E. Björk-Eriksson T. Alborn A. M. Nordborg C. Peterson D. A.. (1998). Neurogenesis in the adult human hippocampus. Nat. Med. 4, 1313–1317. 10.1038/3305 9809557

[B22] Ernst A. Alkass K. Bernard S. Salehpour M. Perl S. Tisdale J.. (2014). Neurogenesis in the striatum of the adult human brain. Cell 156, 1072–1083. 10.1016/j.cell.2014.01.044 24561062

[B23] Flood J. F. Harris F. J. Morley J. E. (1996). Age-related changes in hippocampal drug facilitation of memory processing in SAMP8 mice. Neurobiol. Aging 17, 15–24. 10.1016/0197-4580(95)02007-1 8786798

[B24] Fuster-Matanzo A. de Barreda E. G. Dawson H. N. Vitek M. P. Avila J. Hernández F. (2009). Function of tau protein in adult newborn neurons. FEBS Lett. 583, 3063–3068. 10.1016/j.febslet.2009.08.017 19695252

[B25] Fuster-Matanzo A. Llorens-Martín M. Jurado-Arjona J. Avila J. Hernández F. (2012). Tau protein and adult hippocampal neurogenesis. Front. Neurosci. 6:104. 10.3389/fnins.2012.00104 22787440 PMC3391648

[B26] Gadadhar A. Marr R. Lazarov O. (2011). Presenilin-1 regulates neural progenitor cell differentiation in the adult brain. J. Neurosci. 31, 2615–2623. 10.1523/JNEUROSCI.4767-10.2011 21325529 PMC3050597

[B27] Ge S. Goh E. L. Sailor K. A. Kitabatake Y. Ming G. L. Song H. (2006). GABA regulates synaptic integration of newly generated neurons in the adult brain. Nature 439, 589–593. 10.1038/nature04404 16341203 PMC1420640

[B28] Geinisman Y. Berry R. W. Disterhoft J. F. Power J. M. Van der Zee E. A. (2001). Associative learning elicits the formation of multiple-synapse boutons. J. Neurosci. 21, 5568–5573. 11466428 10.1523/JNEUROSCI.21-15-05568.2001 PMC6762639

[B29] Geinisman Y. Ganeshina O. Yoshida R. Berry R. W. Disterhoft J. F. Gallagher M. (2004). Aging, spatial learning, and total synapse number in the rat CA1 stratum radiatum. Neurobiol. Aging 25, 407–416. 10.1016/j.neurobiolaging.2003.12.001 15123345

[B30] Hamilton L. K. Aumont A. Julien C. Vadnais A. Calon F. Fernandes K. J. (2010). Widespread deficits in adult neurogenesis precede plaque and tangle formation in the 3xTg mouse model of Alzheimer's disease. Eur. J. Neurosci. 32, 905–920. 10.1111/j.1460-9568.2010.07379.x 20726889

[B31] Hattiangady B. Shetty A. K. (2008). Aging does not alter the number or phenotype of putative stem/progenitor cells in the neurogenic region of the hippocampus. Neurobiol. Aging 29, 129–147. 10.1016/j.neurobiolaging.2006.09.015 17092610 PMC3612500

[B32] Herold S. Jagasia R. Merz K. Wassmer K. Lie D. C. (2011). CREB signalling regulates early survival, neuronal gene expression and morphological development in adult subventricular zone neurogenesis. Mol. Cell Neurosci. 46, 79–88. 10.1016/j.mcn.2010.08.008 20801218

[B33] Hong X. P. Peng C. X. Wei W. Tian Q. Liu Y. H. Yao X. Q.. (2010). Essential role of tau phosphorylation in adult hippocampal neurogenesis. Hippocampus 20, 1339–1349. 10.1002/hipo.20712 19816983

[B34] Hu Y. S. Long N. Pigino G. Brady S. T. Lazarov O. (2013). Molecular mechanisms of environmental enrichment: impairments in Akt/GSK3beta, neurotrophin-3 and CREB signaling. PLoS ONE 8:e64460. 10.1371/journal.pone.0064460 23700479 PMC3660250

[B35] Hu Y. S. Xu P. Pigino G. Brady S. T. Larson J. Lazarov O. (2010). Complex environment experience rescues impaired neurogenesis, enhances synaptic plasticity, and attenuates neuropathology in familial Alzheimer's disease-linked APPswe/PS1{Delta}E9 mice. FASEB J. 24, 1667–1681. 10.1096/fj.09-136945 20086049 PMC4050966

[B36] Imayoshi I. Sakamoto M. Ohtsuka T. Takao K. Miyakawa T. Yamaguchi M.. (2008). Roles of continuous neurogenesis in the structural and functional integrity of the adult forebrain. Nat. Neurosci. 11, 1153–1161. 10.1038/nn.2185 18758458

[B37] Jagasia R. Steib K. Englberger E. Herold S. Faus-Kessler T. Saxe M.. (2009). GABA-cAMP response element-binding protein signaling regulates maturation and survival of newly generated neurons in the adult hippocampus. J. Neurosci. 29, 7966–7977. 10.1523/JNEUROSCI.1054-09.2009 19553437 PMC2776747

[B38] Jin K. Peel A. L. Mao X. O. Xie L. Cottrell B. A. Henshall D. C.. (2004). Increased hippocampal neurogenesis in Alzheimer's disease. Proc. Natl. Acad. Sci. U.S.A. 101, 343–347. 10.1073/pnas.2634794100 14660786 PMC314187

[B39] Kandel E. R. (2012). The molecular biology of memory: cAMP, PKA, CRE, CREB-1, CREB-2, and CPEB. Mol. Brain 5:14. 10.1186/1756-6606-5-14 22583753 PMC3514210

[B40] Kang D. E. Soriano S. Xia X. Eberhart C. G. De Strooper B. Zheng H.. (2002). Presenilin couples the paired phosphorylation of beta-catenin independent of axin: implications for beta-catenin activation in tumorigenesis. Cell 110, 751–762. 10.1016/S0092-8674(02)00970-4 12297048

[B41] Kempermann G. Kuhn H. G. Gage F. H. (1997). More hippocampal neurons in adult mice living in an enriched environment. Nature 386, 493–495. 10.1038/386493a0 9087407

[B42] Kempermann G. Song H. Gage F. H. (2015). Neurogenesis in the Adult Hippocampus. Cold Spring Harb. Perspect. Med. 5:a018812. 10.1101/cshperspect.a018812 26330519 PMC4563705

[B43] Klusmann V. Evers A. Schwarzer R. Schlattmann P. Reischies F. M. Heuser I.. (2010). Complex mental and physical activity in older women and cognitive performance: a 6-month randomized controlled trial. J. Gerontol. A Biol. Sci. Med. Sci. 65, 680–688. 10.1093/gerona/glq053 20418350

[B44] Komuro Y. Xu G. Bhaskar K. Lamb B. T. (2015). Human tau expression reduces adult neurogenesis in a mouse model of tauopathy. Neurobiol. Aging 36, 2034–2042. 10.1016/j.neurobiolaging.2015.03.002 25863528 PMC4724414

[B45] Kopan R. Ilagan M. X. G. (2009). The canonical Notch signaling pathway: unfolding the activation mechanism. Cell 137, 216–233. 10.1016/j.cell.2009.03.045 19379690 PMC2827930

[B46] Kudo K. Wati H. Qiao C. Arita J. Kanba S. (2005). Age-related disturbance of memory and CREB phosphorylation in CA1 area of hippocampus of rats. Brain Res. 1054, 30–37. 10.1016/j.brainres.2005.06.045 16054117

[B47] Lange C. Mix E. Frahm J. Glass A. Müller J. Schmitt O.. (2011). Small molecule GSK-3 inhibitors increase neurogenesis of human neural progenitor cells. Neurosci. Lett. 488, 36–40. 10.1016/j.neulet.2010.10.076 21056624

[B48] Lazarov O. Demars M. P. (2012). All in the family: how the APPs regulate neurogenesis. Front. Neurosci. 6:81. 10.3389/fnins.2012.00081 22675290 PMC3366480

[B49] Lazarov O. Hollands C. (2016). Hippocampal neurogenesis: learning to remember. Prog Neurobiol. 138-140:1-18. 10.1016/j.pneurobio.2015.12.006 26855369 PMC4828289

[B50] Lazarov O. Larson J. (2007). Research progress in Alzheimer's disease and dementia, in Environmental Enrichment: from Mouse AD Model to AD Therapy, Vol. 3. (New York, NY: Nova Science Publishers, Inc.), 303–328.

[B51] Lazarov O. Marr R. A. (2010). Neurogenesis and Alzheimer's disease: at the crossroads Exp. Neurol. 223, 267–281. 10.1016/j.expneurol.2009.08.009 19699201 PMC2864344

[B52] Lazarov O. Marr R. A. (2013). Of mice and men: neurogenesis, cognition and Alzheimer's disease. Front. Aging Neurosci. 5:43. 10.3389/fnagi.2013.00043 23986699 PMC3753540

[B53] Lazarov O. Mattson M. P. Peterson D. A. Pimplikar S. W. van Praag H. (2010). When neurogenesis encounters aging and disease. Trends Neurosci. 33, 569–579. 10.1016/j.tins.2010.09.003 20961627 PMC2981641

[B54] Lazarov O. Robinson J. Tang Y. P. Hairston I. S. Korade-Mirnics Z. Lee V. M.. (2005). Environmental enrichment reduces Abeta levels and amyloid deposition in transgenic mice. Cell 120, 701–713. 10.1016/j.cell.2005.01.015 15766532

[B55] Lie D. C. Colamarino S. A. Song H. J. Désiré L. Mira H. Consiglio A.. (2005). Wnt signalling regulates adult hippocampal neurogenesis. Nature 437, 1370–1375. 10.1038/nature04108 16251967

[B56] Llorens-Martin M. Teixeira C. M. Fuster-Matanzo A. Jurado-Arjona J. Borrell V. Soriano E.. (2012). Tau isoform with three microtubule binding domains is a marker of new axons generated from the subgranular zone in the hippocampal dentate gyrus: implications for Alzheimer's disease. J. Alzheimers. Dis. 29, 921–930. 10.3233/JAD-2012-112057. 22337826

[B57] Loewenbruck K. F. Fuchs B. Hermann A. Brandt M. Werner A. Kirsch M.. (2011). Proton MR spectroscopy of neural stem cells: does the proton-NMR peak at 1.28 ppm function as a biomarker for cell type or state? Rejuvenation Res. 14, 371–381. 10.1089/rej.2010.1102 21548757

[B58] Longo F. M. Yang T. Xie Y. Massa S. M. (2006). Small molecule approaches for promoting neurogenesis. Curr. Alzheimer Res. 3, 5–10. 10.2174/156720506775697089 16472196

[B59] Lugert S. Basak O. Knuckles P. Haussler U. Fabel K. Götz M.. (2010). Quiescent and active hippocampal neural stem cells with distinct morphologies respond selectively to physiological and pathological stimuli and aging. Cell Stem Cell 6, 445–456. 10.1016/j.stem.2010.03.017 20452319

[B60] Ma Q. L. Harris-White M. E. Ubeda O. J. Simmons M. Beech W. Lim G. P.. (2007). Evidence of Abeta- and transgene-dependent defects in ERK-CREB signaling in Alzheimer's models. J. Neurochem. 103, 1594–1607. 10.1111/j.1471-4159.2007.04869.x 17760871 PMC2527620

[B61] MacMillan K. S. Naidoo J. Liang J. Melito L. Williams N. S. Morlock L.. (2011). Development of proneurogenic, neuroprotective small molecules. J. Am. Chem. Soc. 133, 1428–1437. 10.1021/ja108211m 21210688 PMC3033481

[B62] Manganas L. N. Zhang X. Li Y. Hazel R. D. Smith S. D. Wagshul M. E.. (2007). Magnetic resonance spectroscopy identifies neural progenitor cells in the live human brain. Science 318, 980–985. 10.1126/science.1147851 17991865 PMC4039561

[B63] Marathe S. Alberi L. (2015). Notch in memories: points to remember. Hippocampus 25, 1481–1488. 10.1002/hipo.22426 25656274

[B64] Marathe S. Liu S. Brai E. Kaczarowski M. Alberi L. (2015). Notch signaling in response to excitotoxicity induces neurodegeneration via erroneous cell cycle reentry. Cell Death Differ. 22, 1775–1784. 10.1038/cdd.2015.23 25822340 PMC4648324

[B65] Mayr B. Montminy M. (2001). Transcriptional regulation by the phosphorylation-dependent factor CREB. Nat. Rev. Mol. Cell Biol. 2, 599–609. 10.1038/35085068 11483993

[B66] McNeish J. Roach M. Hambor J. Mather R. J. Weibley L. Lazzaro J.. (2010). High-throughput screening in embryonic stem cell-derived neurons identifies potentiators of alpha-amino-3-hydroxyl-5-methyl-4-isoxazolepropionate-type glutamate receptors. J. Biol. Chem. 285, 17209–17217. 10.1074/jbc.M109.098814 20212047 PMC2878023

[B67] Merz K. Herold S. Lie D. C. (2011). CREB in adult neurogenesis–master and partner in the development of adult-born neurons? Eur. J. Neurosci. 33, 1078–1086. 10.1111/j.1460-9568.2011.07606.x 21395851

[B68] Neely M. D. Litt M. J. Tidball A. M. Li G. G. Aboud A. A. Hopkins C. R.. (2012). DMH1, a highly selective small molecule BMP inhibitor promotes neurogenesis of hiPSCs: comparison of PAX6 and SOX1 expression during neural induction. ACS Chem. Neurosci. 3, 482–491. 10.1021/cn300029t 22860217 PMC3400384

[B69] Ninkovic J. Götz M. (2007). Signaling in adult neurogenesis: from stem cell niche to neuronal networks. Curr. Opin. Neurobiol. 17, 338–344. 10.1016/j.conb.2007.04.006 17475475

[B70] Ortega-Martínez S. (2015). A new perspective on the role of the CREB family of transcription factors in memory consolidation via adult hippocampal neurogenesis. Front. Mol. Neurosci. 8:46. 10.3389/fnmol.2015.00046 26379491 PMC4549561

[B71] Ozgen N. Lau D. H. Shlapakova I. N. Sherman W. Feinmark S. J. Danilo P. Jr.. (2010). Determinants of CREB degradation and KChIP2 gene transcription in cardiac memory. Heart Rhythm 7, 964–970. 10.1016/j.hrthm.2010.03.024 20346417 PMC2904822

[B72] Petrik D. Jiang Y. Birnbaum S. G. Powell C. M. Kim M. S. Hsieh J.. (2012). Functional and mechanistic exploration of an adult neurogenesis-promoting small molecule. FASEB J. 26, 3148–3162. 10.1096/fj.11-201426 22542682 PMC3405259

[B73] Pieper A. A. Xie S. Capota E. Estill S. J. Zhong J. Long J. M.. (2010). Discovery of a proneurogenic, neuroprotective chemical. Cell 142, 39–51. 10.1016/j.cell.2010.06.018 20603013 PMC2930815

[B74] Pierfelice T. Alberi L. Gaiano N. (2011). Notch in the vertebrate nervous system: an old dog with new tricks. Neuron 69, 840–855. 10.1016/j.neuron.2011.02.031 21382546

[B75] Porte Y. Buhot M. C. Mons N. (2008). Alteration of CREB phosphorylation and spatial memory deficits in aged 129T2/Sv mice. Neurobiol. Aging 29, 1533–1546. 10.1016/j.neurobiolaging.2007.03.023 17478013

[B76] Rodríguez J. J. Jones V. C. Tabuchi M. Allan S. M. Knight E. M. LaFerla F. M.. (2008). Impaired adult neurogenesis in the dentate gyrus of a triple transgenic mouse model of Alzheimer's disease. PLoS ONE 3:e2935. 10.1371/journal.pone.0002935 18698410 PMC2492828

[B77] Ruscheweyh R. Willemer C. Krüger K. Duning T. Warnecke T. Sommer J.. (2011). Physical activity and memory functions: an interventional study. Neurobiol. Aging 32, 1304–1319. 10.1016/j.neurobiolaging.2009.08.001 19716631

[B78] Sahay A. Scobie K. N. Hill A. S. O'Carroll C. M. Kheirbek M. A. Burghardt N. S.. (2011). Increasing adult hippocampal neurogenesis is sufficient to improve pattern separation. Nature 472, 466–470. 10.1038/nature09817 21460835 PMC3084370

[B79] Sato N. Meijer L. Skaltsounis L. Greengard P. Brivanlou A. H. (2004). Maintenance of pluripotency in human and mouse embryonic stem cells through activation of Wnt signaling by a pharmacological GSK-3-specific inhibitor. Nat. Med. 10, 55–63. 10.1038/nm979 14702635

[B80] Schneider J. W. Gao Z. Li S. Farooqi M. Tang T. S. Bezprozvanny I.. (2008). Small-molecule activation of neuronal cell fate. Nat. Chem. Biol. 4, 408–410. 10.1038/nchembio.95 18552832

[B81] Scott Bitner R. (2012). Cyclic AMP response element-binding protein (CREB) phosphorylation: a mechanistic marker in the development of memory enhancing Alzheimer's disease therapeutics. Biochem. Pharmacol. 83, 705–714. 10.1016/j.bcp.2011.11.009 22119240

[B82] Selkoe D. J. Wolfe M. S. (2007). Presenilin: running with scissors in the membrane. Cell 131, 215–221. 10.1016/j.cell.2007.10.012 17956719

[B83] Shi J. Longo F. M. Massa S. M. (2013). A small molecule p75(NTR) ligand protects neurogenesis after traumatic brain injury. Stem Cells 31, 2561–2574. 10.1002/stem.1516 23940017

[B84] Shimizu T. Kagawa T. Inoue T. Nonaka A. Takada S. Aburatani H.. (2008). Stabilized beta-catenin functions through TCF/LEF proteins and the Notch/RBP-Jkappa complex to promote proliferation and suppress differentiation of neural precursor cells. Mol. Cell Biol. 28, 7427–7441. 10.1128/MCB.01962-07 18852283 PMC2593432

[B85] Silva-Vargas V. Crouch E. E. Doetsch F. (2013). Adult neural stem cells and their niche: a dynamic duo during homeostasis, regeneration, and aging. Curr. Opin. Neurobiol. 23, 935–942. 10.1016/j.conb.2013.09.004 24090877

[B86] Spalding K. Bergmann O. Alkass K. Bernard S. Salehpour M. Huttner H.. (2013). Dynamics of hippocampal neurogenesis in adult humans Cell. 153, 1219–1227 10.1016/j.cell.2013.05.002 23746839 PMC4394608

[B87] Stark S. M. Yassa M. A. Stark C. E. (2010). Individual differences in spatial pattern separation performance associated with healthy aging in humans. Learn. Mem. 17, 284–288. 10.1101/lm.1768110 20495062 PMC2884287

[B88] Sun B. Halabisky B. Zhou Y. Palop J. J. Yu G. Mucke L.. (2009). Imbalance between GABAergic and glutamatergic transmission impairs adult neurogenesis in an animal model of Alzheimer's Disease. Cell Stem Cell 5, 624–633. 10.1016/j.stem.2009.10.003 19951690 PMC2823799

[B89] Tesco G. Kim T. W. Diehlmann A. Beyreuther K. Tanzi R. E. (1998). Abrogation of the presenilin 1/beta-catenin interaction and preservation of the heterodimeric presenilin 1 complex following caspase activation. J. Biol. Chem. 273, 33909–33914. 10.1074/jbc.273.51.33909 9852041

[B90] Tesco G. Tanzi R. E. (2000). GSK3 beta forms a tetrameric complex with endogenous PS1-CTF/NTF and beta-catenin. Effects of the D257/D385A and FAD-linked mutations. Ann. N.Y. Acad. Sci. 920, 227–232. 10.1111/j.1749-6632.2000.tb06927.x 11193155

[B91] Toner C. K. Pirogovsky E. Kirwan C. B. Gilbert P. E. (2009). Visual object pattern separation deficits in nondemented older adults. Learn. Mem. 16, 338–342. 10.1101/lm.1315109 19403797

[B92] Tseng C. Y. Kao S. H. Wan C. L. Cho Y. Tung S. Y. Hsu H. J. (2014). Notch signaling mediates the age-associated decrease in adhesion of germline stem cells to the niche. PLoS Genet. 10:e1004888. 10.1371/journal.pgen.1004888 25521289 PMC4270478

[B93] van Praag H. Christie B. R. Sejnowski T. J. Gage F. H. (1999a). Running enhances neurogenesis, learning, and long-term potentiation in mice. Proc. Natl. Acad. Sci. U.S.A. 96, 13427–13431. 10.1073/pnas.96.23.13427 10557337 PMC23964

[B94] van Praag H. Kempermann G. Gage F. H. (1999b). Running increases cell proliferation and neurogenesis in the adult mouse dentate gyrus [see comments]. Nat. Neurosci. 2, 266–270. 10.1038/6368 10195220

[B95] Villeda S. A. Luo J. Mosher K. I. Zou B. Britschgi M. Bieri G.. (2011). The ageing systemic milieu negatively regulates neurogenesis and cognitive function. Nature 477, 90–94. 10.1038/nature10357 21886162 PMC3170097

[B96] Villeda S. A. Wyss-Coray T. (2013). The circulatory systemic environment as a modulator of neurogenesis and brain aging. Autoimmun. Rev. 12, 674–677. 10.1016/j.autrev.2012.10.014 23201925

[B97] Vitolo O. V. Sant'Angelo A. Costanzo V. Battaglia F. Arancio O. Shelanski M. (2002). Amyloid beta -peptide inhibition of the PKA/CREB pathway and long-term potentiation: reversibility by drugs that enhance cAMP signaling. Proc. Natl. Acad. Sci. U.S.A. 99, 13217–13221. 10.1073/pnas.172504199 12244210 PMC130613

[B98] Wahlster L. Arimon M. Nasser-Ghodsi N. Post K. L. Serrano-Pozo A. Uemura K.. (2013). Presenilin-1 adopts pathogenic conformation in normal aging and in sporadic Alzheimer's disease. Acta Neuropathol. 125, 187–199. 10.1007/s00401-012-1065-6 23138650 PMC3552123

[B99] Wang R. Dineley K. T. Sweatt J. D. Zheng H. (2004). Presenilin 1 familial Alzheimer's disease mutation leads to defective associative learning and impaired adult neurogenesis. Neuroscience 126, 305–312. 10.1016/j.neuroscience.2004.03.048 15207348

[B100] Wang W. Pan Y. W. Zou J. Li T. Abel G. M. Palmiter R. D.. (2014). Genetic activation of ERK5 MAP kinase enhances adult neurogenesis and extends hippocampus-dependent long-term memory. J. Neurosci. 34, 2130–2147. 10.1523/JNEUROSCI.3324-13.2014 24501354 PMC3913867

[B101] Warner-Schmidt J. L. Duman R. S. (2007). VEGF is an essential mediator of the neurogenic and behavioral actions of antidepressants. Proc. Natl. Acad. Sci. U.S.A. 104, 4647–4652. 10.1073/pnas.0610282104 17360578 PMC1838655

[B102] Wen P. H. Hof P. R. Chen X. Gluck K. Austin G. Younkin S. G.. (2004). The presenilin-1 familial Alzheimer disease mutant P117L impairs neurogenesis in the hippocampus of adult mice. Exp. Neurol. 188, 224–237. 10.1016/j.expneurol.2004.04.002 15246822

[B103] Xia D. Watanabe H. Wu B. Lee S. H. Li Y. Tsvetkov E.. (2015). Presenilin-1 knockin mice reveal loss-of-function mechanism for familial Alzheimer's disease. Neuron 85, 967–981. 10.1016/j.neuron.2015.02.010 25741723 PMC4358812

[B104] Xia X. Qian S. Soriano S. Wu Y. Fletcher A. M. Wang X. J.. (2001). Loss of presenilin 1 is associated with enhanced beta-catenin signaling and skin tumorigenesis. Proc. Natl. Acad. Sci. U.S.A. 98, 10863–10868. 10.1073/pnas.191284198 11517342 PMC58565

[B105] Yassa M. A. Mattfeld A. T. Stark S. M. Stark C. E. (2011). Age-related memory deficits linked to circuit-specific disruptions in the hippocampus. Proc. Natl. Acad. Sci. U.S.A. 108, 8873–8878. 10.1073/pnas.1101567108 21555581 PMC3102362

[B106] Young-Pearse T. L. Bai J. Chang R. Zheng J. B. LoTurco J. J. Selkoe D. J. (2007). A critical function for beta-amyloid precursor protein in neuronal migration revealed by in utero RNA interference. J. Neurosci. 27, 14459–14469. 10.1523/JNEUROSCI.4701-07.2007 18160654 PMC6673432

[B107] Young-Pearse T. L. Chen A. C. Chang R. Marquez C. Selkoe D. J. (2008). Secreted APP regulates the function of full-length APP in neurite outgrowth through interaction with integrin beta1. Neural Dev. 3:15. 10.1186/1749-8104-3-15 18573216 PMC2442059

